# Educational impact of Mini-Clinical Evaluation Exercise: A game changer

**DOI:** 10.12669/pjms.342.14667

**Published:** 2018

**Authors:** Lamia Yusuf, Amina Ahmed, Raheela Yasmin

**Affiliations:** 1 Dr. Lamia Yusuf MBBS, FCPS, MHPE. Assistant Professor (Gynaecology / Obstetrics) Rashid Latif Medical College, Lahore, Pakistan.; 2 Dr. Amina Ahmed, MBBS, M. PHIL, MCHP-HPE. Supervisor, Associate Professor, Medical Education, Fatima Memorial College, Lahore, Pakistan; 3 Prof. Raheela Yasmin, BDS, DCPS-HPE, MHPE, PHD-HPE. Co-supervisor, Medical Education, Riphah international University, Islamabad, Pakistan

**Keywords:** Work place-based assessment, Educational impact, Minicex

## Abstract

**Background and objective:**

Workplace based assessment has a strong educational impact in terms of student’s clinical performance by steering their learning towards the desired learning outcomes. Educational impact is hardly measured in the sphere of medical education and this study is an attempt to measure educational impact of post graduate residents. The aim of this study was “To explore educational impact of Minicex (Mini-clinical evaluation exercise) on residents with respect to their learning”.

**Methods:**

A mixed convergent parallel method was selected, participants were identified through non-probability convenience sampling, total 10 participants were chosen for data collection, all of them experienced four minicex encounters which generated their scores (the quantitative data), after first two Minicex encounters each participant was interviewed using a structured interview technique, similarly, after 3^rd^ and 4^th^ Minicex encounters. Data was entered in the SPSS version -21 to calculate descriptive statistics. Inferential statistics were determined using ANOVA to calculate improvement in score over time and P-value to report statistical significance. Qualitative analysis was done using thematic analysis approach with the help of themes based on interview questions: priori theme. NVIVO was used for triangulation of themes accordingly.

**Results:**

The results indicate statistically significant improvement in scores and p values were considered significant at 0.05. Also, qualitative analysis provided reasons for improvement in scores and residents’ satisfaction such as feedback, motivation, self-directed learning, peer assisted learning.

**Conclusion:**

The study concluded that residents learning behavior and, their satisfaction from assessment method can be enhanced through work place based assessment particularly in context of minicex (mini-clinical evaluation exercise) so encouraging its use in similar situations. However, the scope for generalization of results remains limited owing to a small sample size.

## INTRODUCTION

Educational impact is the impact on students learning, which causes change in behavior of learner. It determines consequential validity of an assessment. Students learning and student’s behavior are very much influenced by assessments and their consequential validity, both factors play a vital role in shaping up student’s behaviors. Never the less Workplace based assessment(WPBA) have emerged as an important element in this development, targeting to assess on the highest level of Miller’s pyramid the does level.^1^ WPBA defined by GMC is assessment of working practices, based on what trainees actually do in their work place.^2^ WPBA includes direct observation of trainee’s performances at his/her workplace followed by provision of feedback on different domains.^3^ The primary purpose of WPBA is to help learners by providing structured feedback (assessment for learning). Feedback is hallmark of formative assessment,^4^ which directs learning toward desired direction thus ensuring a desirable consequential validity and therefore positive educational impact of assessment. One of the most important benefit of WPBA is one to one encounter of evaluator and trainee, and the feedback is generated immediately. Feed-back can change clinical performances when it is systematically given by credible sources. However, implementation of WBA is challenging.^5^ One of the assessment tool for WPBA is Minicex (mini clinical evaluation exercise). It is used for assessing clinical skills, and it is meant for formative assessment. The reason for selecting Minicex as a WPBA tool in research is that it has many advantages, it is relatively of shorter duration, it can take place in a variety of clinical settings, almost immediate feedback is being given , its validity and reliability is better. In addition to this trainee are exposed to variety of assessors which expose them to different viewpoints, which is a strength of this method.^6^

Although evidence of educational impact of formative assessment is available outside the sphere of medical education, but concrete evidence of formative assessment or work place based assessment is lacking in medical education.^1^,^5^,^7^,^8^ It has been observed that during residency programs for fellowship training by College of Physician & Surgeon Pakistan, hardly any feedback is given to trainee, and neither they are formally observed. Though CPSP since 2009 after introduction of e log book, has given Supervisors the opportunity to give feedback about their trainees, and trainees are asked to maintain their portfolio.^9^ However, poor implementation across the country, indicates need for a more focused approach to improve training. Using Mini-cex for assessment for learning of (Fellow college of physician & surgeon Pakistan) trainees may generate evidence supporting desirable educational impact of the tool. This may facilitate transferability of these findings to similar contexts in Pakistan and thus implementation of Mini-CEX.^7^,^10^

## METHODS

The study period was of six months duration. The Participants were recruited from Rashid Latif Medical College Lahore. All of them were residents of gynecology/obstetrics. The ethical approval was obtained from the ethical review committee. A mixed method convergent / parallel or concurrent mixed method design was used.^11^ Both quantitative and qualitative data were collected simultaneously. Both data were analyzed separately, and results were triangulated accordingly in the discussion section to demonstrate plausibility and confirmability of interpretations. In all 10 post-graduate students were selected by non-probability convenience sampling, to collect data. (The principal researcher is not supervisor of CPSP). Director Department of medical education send request to all residents to participate in study and those who were willing for participation were added in this study. We started data collection with 10 participants for both mini-cex encounters and in-depth interviews. We were cognizant of the fact that addition of more participants might have been needed, if data saturation could not be achieved, with 10 participants. However, data saturation was achieved with the initial 10 participants, hence data collection was terminated.^11^ In total, 40 Minicex encounters were performed and 20 structured interviews were conducted. A written consent was taken from participants regarding their participation in this research.^12^ We sought permission from ABIM(American Board of internal medicine) to use this rating scale for research purposes. All seven components^4^ of Minicex rating scale including: history taking, physical examination, communication skills, clinical judgement, professionalism, organization/efficiency and overall clinical judgement were scored.^10^ Performance was rated on 9-point scale, where 1,2,3 indicated: unsatisfactory performance, 4: marginal performance, 5, 6: satisfactory, 7, 8 and 9: superior performances.^9^

Structured interview with pre-determined open ended questions in pre- determined sequence were conducted on one-on-one basis.^10^,^12^ Interview protocols were designed, which included instructions for the process of interview, the questions to be asked, probes, and prompts to be used and space to take field notes of responses from the interviewees. Participants were informed 48 hours before their Minicex examination. They were sent interview questions and interview protocols before hand for better familiarization with the procedure.^11^,^12^ Each encounter lasted for 15-20 minutes. Feedback and ratings were given immediately to the participants by principal researcher and each feedback lasted for 5-10 minutes. After two Minicex encounters, the principal researcher took first semi-structured interview. Interviews were audio recorded. After first interview, each participant experienced two more Minicex encounters and were rated accordingly. Same process of interviewing was repeated after two more minicex encounters. Each interview started with an open-ended question “How did you prepare for this assessment?” It was followed by a few probes. No leading questions were asked to avoid biased opinions. As far as possible, honest narrative of participants was obtained. Each participant was given a pseudonym to maintain confidentiality during transcription and subsequent analysis.

***Quantitative data analysis*** the numerical data was collected through Minicex rating scales (in terms of trainee’s ratings). Data was entered in the SPSS version -21 to calculate descriptive statistics such as: mean, SD, frequencies, and histogram. Inferential statistics were determined using ANOVA to calculate improvement in score over time and P-value to report statistical significance.

***Qualitative data analysis*** the interviews were transcribed verbatim by the principal researcher and given to participants/residents for verification of responses. Priori themes were identified from the interview questions and were refined by reviewing the transcripts. The transcribed data was entered N-Vivo 11. It generated, word analysis for each theme and word cloud, vindicating triangulation with manually generated priori themes and comments verbatim, quoted under each theme. Constant iterative approach was used to compare the data analyzed and inferences drawn with the research question through member checking to ensure plausibility, confirmability and sturdiness of results.^11^,^12^

## RESULTS

In this study ten subjects were enrolled. All the participants were female and as the setting was gynecology department therefore the patients were also female. The continuous variables were expressed as means and Standard deviations using Post hoc Tukey’s test. The categorical variables were summarized as proportions. A p value of less than 0.05 was taken statistically significant a repeated measures analysis of variance ANOVA was conducted. ALL data were analyzed using SPSS 21, to determine improvement in scores over time (Table-I). The Normality of data was tested by drawing histograms. Shaprio -wilk test was applied, with a p-value of< 0.05 taken as statistically significant. Thematic analysis identified seven themes shown in Table-II.

**Table-I:** Statistical analysis of all components of minicex.

	Minicex encounters	N	Mean	Std. Deviation	Std. Error	95% Confidence Interval for Mean	

						Lower Bound	Upper Bound
Medical Interviewing Skills	1.00	10	3.7000	1.05935	.33500	2.9422	4.4578
	2.00	10	5.2000	1.03280	.32660	4.4612	5.9388
	3.00	10	6.4000	.69921	.22111	5.8998	6.9002
	4.00	10	7.1000	.73786	.23333	6.5722	7.6278
	**Total**	40	5.6000	1.56566	.24755	5.0993	6.1007
Physical Examination Skills	1.00	10	4.0000	.81650	.25820	3.4159	4.5841
	2.00	10	5.3000	.67495	.21344	4.8172	5.7828
	3.00	10	6.3000	.82327	.26034	5.7111	6.8889
	4.00	10	7.5000	1.26930	.40139	6.5920	8.4080
	**Total**	40	5.7750	1.57688	.24933	5.2707	6.2793
Humanistic Qualities/ Professionalism	1.00	10	4.2000	1.03280	.32660	3.4612	4.9388
	2.00	10	5.4000	.69921	.22111	4.8998	5.9002
	3.00	10	6.4000	.69921	.22111	5.8998	6.9002
	4.00	10	7.2000	.91894	.29059	6.5426	7.8574
	**Total**	40	5.8000	1.39963	.22130	5.3524	6.2476
Clinical Judgment	1.00	10	4.1000	.99443	.31447	3.3886	4.8114
	2.00	10	5.4000	.69921	.22111	4.8998	5.9002
	3.00	10	6.6000	1.07497	.33993	5.8310	7.3690
	4.00	10	7.6000	1.26491	.40000	6.6951	8.5049
	**Total**	40	5.9250	1.65464	.26162	5.3958	6.4542
Counselling Skills	1.00	9	3.8889	1.16667	.38889	2.9921	4.7857
	2.00	10	5.1000	.87560	.27689	4.4736	5.7264
	3.00	10	6.5000	.70711	.22361	5.9942	7.0058
	4.00	10	7.8000	1.31656	.41633	6.8582	8.7418
	**Total**	39	5.8718	1.77970	.28498	5.2949	6.4487
Organization/ Efficiency	1.00	10	3.9000	1.19722	.37859	3.0436	4.7564
	2.00	10	5.7000	.67495	.21344	5.2172	6.1828
	3.00	10	6.4000	.96609	.30551	5.7089	7.0911
	4.00	10	7.5000	1.35401	.42817	6.5314	8.4686
	**Total**	40	5.8750	1.68230	.26599	5.3370	6.4130
Overall Clinical Competence	1.00	8	4.6250	1.06066	.37500	3.7383	5.5117
	2.00	10	5.7000	.48305	.15275	5.3544	6.0456
	3.00	10	7.0000	1.15470	.36515	6.1740	7.8260
	4.00	10	7.6000	1.26491	.40000	6.6951	8.5049
	**Total**	38	6.3158	1.50863	.24473	5.8199	6.8117
Evaluator Satisfaction with Mini-CEX	1.00	3	4.0000	1.00000	.57735	1.5159	6.4841
	2.00	6	5.8333	.40825	.16667	5.4049	6.2618
	3.00	8	6.7500	1.16496	.41188	5.7761	7.7239
	4.00	10	7.5000	.97183	.30732	6.8048	8.1952
	**Total**	27	6.5185	1.42425	.27410	5.9551	7.0819
Resident Satisfaction with Mini-CEX	1.00	1	5.0000	.	.	.	.
	2.00	6	6.0000	1.09545	.44721	4.8504	7.1496
	3.00	6	7.0000	1.26491	.51640	5.6726	8.3274
	4.00	10	7.4000	1.17379	.37118	6.5603	8.2397
	**Total**	23	6.8261	1.30217	.27152	6.2630	7.3892

Qualitative analysis: Following themes and sub themes were identified.

**Table-II:** Thematic analysis.

Themes	Interview 1	Interview 2
Evolution of expectations	“I was expecting that I am going to be assessed on my clinical grounds, how I take my history, how I do examination, how I gain my patient confidence and counsel and diagnose her diseases and how she is treated.”	“After first mini CEX I was expecting that my theoretical knowledge about history taking is quite deficient so I go through different knowledge sources. I have also go through different clips on you tube about physical examination, each mini CEX improve a lot my performance.”
Feed back	“Positive feedback efficiently improved my learning”.	“Feedback is the only thing that tells us about the weak points in your performance so following the feedback of seniors, we could overcome our deficiencies and further improve our good points and then perform better in future.”
Peer Assisted Learning	“Watching friends attempting the Mini CEX helped a lot and I learned from their mistakes and their experience and try it not to repeat those mistakes”.	“Also discussed with my colleagues and then I also consulted with my seniors and with their mutual discussion we try to improved our self-more”.
Factors responsible for learning Confidence Self-motivation	“It was very helpful it improves or increase my morale and my confidence level so that I can perform well in future”	“As I compare my first mini CEX and now at this position I am very confident that I can do a good examination and take a good history I can organize all the points of the history and I can present a good case in front of my seniors”. “Now I can do something, I have polished my abilities to deal the patients and do much more good in clinical practice.”
Learning process		“My performance improves from the 1st 2nd and 3rd and now in 4th Mini CEX. In my first Mini CEX my score was not good. But I practice it again and again and by appearing in Minicex again and again I improve a lot in my history taking physical examination in patient’s approach and in gaining the patients confidence my professionalism and my clinical assessment also improve. So, it was very helpful exercise.”
Knowledge		I am better prepared for theory paper, because for history and physical examination and to go in front of patients, we need to have a lot of knowledge for our clinical assessment. So, I go through the books before appearing in the Mini CEX. So, I have prepared many topics regarding the theory.”
Reduced examination fear	“In exams, if we are giving it first time we are very nervous and disturb, when we do it daily practice it reduces the fear of exams and we do it more confidently and efficiently and with more determination”.	

## DISCUSSION

Educational impact determines quality of student’s learning and learning can be both change in behavior and construction of new knowledge. The discussion shared both quantitative (Table-I) and qualitative (Table-II) results, thus triangulated accordingly. Evolution of expectations indicated educational impact of Minicex, which improved with the passage of time and resulted in change in preparation style for the subsequent mini-cex encounters [comments verbatim Table-II]. Initially participants had bookish knowledge^11^ and then it became more conceptual as residents were engaged in practice sessions. It was found that 21.43% of the participants prepared from books, same was in other study by Tokode & Dennick, 2013.^8^

Similarly, 14.29% of the participants prepared through practicing history taking skills to improve their scores, which is actually desirable consequential validity of Minicex. Formative nature of Minicex promotes deep learning among learners. During evolution of expectations and modification in behaviors, participants prepared themselves for these minicex encounters by practicing in wards and OPDS. Wards and OPDS are similar to the real world where participants will work in future, thus practicing at the DOES level. According to our findings, residents correlate with their mistakes and read topics accordingly to prepare for next minicex. They try to avoid those mistakes again & again. Similar findings were found in study by Foley T et al.^13^ In this study, the participants describe change in their learning behavior and strategies owing to educational impact of an assessment.

Thematic analysis shows Feedback was found to be helpful for 21.43% of the participants, who prepared in light of rater’s feedback. These findings were similar to the findings described in study by Malhotra et al,^14^ which stated that feedback provided by the assessors produced insights among residents regarding their strengths and weaknesses in clinical skills. Feedback means observing an event, critical appraisal of the event according to the standards set, and suggestions for improvement.^15^ The scores show gradual improvement as participants experienced more encounters. Later on, during interviews, they strongly endorsed feedback as reason for improvement in learning.^16^

Through Peer Assisted Learning, participants modify, extend and rebuild their knowledge, develop and share understanding, receive feedback and show reinforcement. This may lead to change in behavior and learning.^17^,^18^ These findings are consistent with our study, 14.29% of the participant were of the opinion that (PAL) was the reason for improvement in scores and change in learning strategies (Table-II). Practice and repetition of components of minicex in daily practice was strategy of 14.29% of the participants (Table-II). Self-motivation and motivation given by others played a vital role in stimulating participants for further minicex encounters. Same findings were observed in another study by Cobb Ka et al.^19^ It implies that student approach to learning process is largely influenced by motivation, confidence and many other environmental factors. Clinical places are social – cultural communities of practices. Sociocultural theories of workplace learning claim that learning and learning outcomes evolve through active participation in activities and in interaction with dynamic systems of the clinical work environment without the fear of being judged or penalized, hence helping in fostering a community of practice,^20^ and reducing fear of performing in real word. Almost all of the participants acknowledged that their fear for examination has been reduced after going through these Minicex encounters same fact addressed in Malhotra et al.^14^ Although the purpose of this research was not preparation of examination, but somehow candidates thought that these encounters prepared them for part 2 exams.^21^ Our study statistically determined residents’ satisfaction with their learning and with Minicex examination. It reflects resident’s satisfaction toward exam format, results, and their performance (Table-I). This fact was very well articulated while addressing the interview questionnaire,^22^ thus achieving resident’s satisfaction in the light of Kirkpatrick evaluation model application at level 2. Qualitative and Quantitive results indicate that the first and second levels of Kirkpatrick evaluation model have been achieved in our study in terms of change in behavior, satisfaction (comment verbatim Table-II) and improvement in scores.^23^,^24^

### Limitations of the study

It was performed on only residents of obstetrics & gynecology. It is suggested to expand this study on other specialties as well. One of the major limitations was limited number of participants were involved to address red tapeism regarding gate keepers’ permission. In addition, qualitative portion needed in-depth exploration. Hence the rule of thumb for sample size for qualitative studies “less is more” was adopted. However, literature is full of quantitive study in Minicex context for generalization. Therefore, more qualitative studies may be done in different specialties. It did not find relationship between learning styles, learning strategy and level of trainings of participants. It did not report the effect of learning of residents in a real -world setting. It could not determine patients’ satisfaction which should have been recorded after change in resident’s behavior. Finally, CPSP is encouraged to introduce Minicex as regular feature of formative assessment during residency years, so as to enhance the educational impact of their assessments.

## CONCLUSION

The study concludes that residents learning behavior, motivation to learn clinical skills and satisfaction from assessment method can all be modified through workplace based assessments, (with special reference to Minicex). Assessments truly drive learning and is established through our study as well. If the assessment measures day to day clinical cases residents start taking interest mastering those cases. In the process of mastery acquisition, they learn all those skills which are relevant and essential such as; medical interviewing skills, physical examination skills, counselling skills, humanistic/professionalism skills, organization skills, clinical judgement skills and overall organization skills.

### Authors’ Contribution

***Lamia Yusuf*** Conceived the idea, did literature review, data collection, analysis and manuscript writing.

***Amina Ahmed*** Main supervisor, involved in every step of research, contributed in discussion. Review and analyzed the final manuscript

***Raheela Yasmin*** Co- supervisor, involved in each step of research and final analysis.

***Lamia Yusuf*** Takes the responsibility and is accountable for all aspects of the work in ensuring that questions related to the accuracy or integrity of any part of the work are appropriately investigated and resolved.
